# A Simple ICT-Based Fluorescent Probe for HOCl and Bioimaging Applications

**DOI:** 10.3390/bios13070744

**Published:** 2023-07-18

**Authors:** Yan Zheng, Shuang Wu, Yifan Bing, Huimin Li, Xueqin Liu, Wenlan Li, Xiang Zou, Zhongyuan Qu

**Affiliations:** 1 School of Pharmacy, Harbin University of Commerce, Harbin 150076, China; 2 Engineering Research Center on Natural Antineoplastic Drugs, Ministry of Education, Harbin University of Commerce, Harbin 150076, China

**Keywords:** bioimaging, hypochlorous acid, fluoxetine, drug-induced liver injury, fluorescence probe

## Abstract

Over the past few decades, drug-induced liver damage (DILI) has become a serious public health problem due to drug abuse. Among multifarious reactive oxygen species, mounting evidence attests that ClO^−^ has been used as a potential biomarker in DILI. In this work, a new “turn-on” fluorescent probe **1** was designed and synthesized by modifying 4′-hydroxybiphenyl-4-carbonitrile (dye **2**) with *N, N*-dimethylthiocarbamate as a response site for detecting ClO^−^. Probe **1** displayed a low detection limit (72 nM), fast response time (30 s), wide pH operating range (6–8), great tissue penetration, large Stokes shift (125 nm) and 291-fold fluorescence enhancement at 475 nm in the mapping of ClO^−^. Probe **1** could trace amounts of exogenous and endogenous ClO^−^ with high sensitivity in MCF-7 cells and HeLa cells. Expectantly, the fluoxetine-induced liver injury model is successfully established, and probe **1** has been used for detecting the fluctuation of ClO^−^ levels in the mouse model of fluoxetine-induced liver injury. All in all, probe **1** with its high specificity, good biological compatibility and liver tissue penetration ability is expected to assist with the early diagnosis of DILI and the clinical screening of various new drugs. We expect that probe **1** could be efficiently used as a powerful molecular tool to predict clinical DILI and explore molecular mechanisms between molecules and disease.

## 1. Introduction

Hypochloric acid (HClO) is known as an extremely important reactive oxygen species (ROS) and endogenous messenger in various physiological processes [1,2,3]. Cellular HClO is mainly generated by the biological oxidation reaction of hydrogen peroxide (H_2_O_2_) and chloride ions (Cl^−^) induced by reduced nicotinamide adenine dinucleotide phosphate (NADPH) on the mitochondrial membrane of neutrophils, which is catalyzed by myeloperoxidase (MPO) [4]. HClO is a crucial component in the innate immune system of living organisms [5]. Under normal circumstances, the production and elimination of HClO in the organism is a process of dynamic balance, whereas higher accumulation levels of exogenous stimulation trigger HClO overproduction [6,7,8]. This imbalance of excessive HClO can cause oxidative damage to DNA and RNA, inflammation and a series of diseases [9]. Recent biomedical research have confirmed that the oxidative damage of host tissue is tightly associated with the aberrant content of HClO, suggesting that HClO dependency could be employed as a potential indicator to understanding the early detection drug-induced liver injury (DILI) [10]. Nevertheless, drug-induced liver injury is an insidious disease. Although the previous organic small molecule fluorescent probes can satisfactorily detect the various markers (ALP, ClO^−^, ONOO^−^, NO, H_2_O_2_) related to the generation of DILI, the development on the detection of DILI by the fluorescence imaging technology is still in the early stage. Unfortunately, because the toxicological investigations of these small molecule probes were not mentioned, the research of DILI by these probes has only been studied at the cellular level. Therefore, it is important to develop tools for the quantitative analysis of HClO in biosystems to clarify its pathophysiology role in vivo.

Up to now, various methods have been established for the detection of ClO^−^, such as high performance liquid chromatography, chemiluminescence and colorimetric methods [11,12,13]. Although these technological means can be applied for the detection and analysis of ClO^−^, the shortcomings, including its cumbersome sample processing, long detection time, and consumables with high cost, limit their further application in the real biological environment. Nevertheless, fluorescence imaging technology has been became one of the most useful tools and it is widely used in chemical testing, biological analysis and medical research owing to its outstanding advantages such as high sensitivity, specific selectivity, and real-time non-invasiveness. At present, by means of the oxidation reactions, a series of organic small molecule fluorescence probes for ClO^−^ detection have been established (the typical cases are displayed in Table S1) [14,15,16,17,18,19,20,21,22,23], including boron dipyrromethene, rhodamine, naphthalimide, triphenylamine, coumarin and nile red. Those dyes were modified with a variety of ClO^−^ specific recognition sites to form the different kinds of organic small molecule fluorescent probes. The synthetic fluorescent probes released these dyes by reacting with ClO^−^ so as to achieve the sensing of HClO with the optical signal of different fluorescence channels. In spite of great progress in ClO^−^ sensing, few probes are applied to monitor and image antidepressant-induced ClO^−^ in liver injury. In addition, the exploration of fluorescence probes with weak initial fluorescence and high fluorescence quantum yield, high specificity, large Stokes shift and excellent fluorescence enhancement is still a focus in scientific research [24,25,26,27,28,29,30,31,32]. Thus, developing a fluorescence probe with effective sensitivity for ClO^−^ visualization in vivo and antidepressant-damaged liver tissues is of great practical value.

As an ICT (Intramolecular Charge Transfer)-based fluorescent dye, 4′-hydroxybiphenyl-4-carbonitrile (dye **2**) has many desirable features, which are attributed to its great push–pull electronic system, such as emission in the cyan spectral region and good photo-stabilities [33]. In addition, the *N, N*-dimethylthiocarbamate group has been widely used as a specific response site for ClO^−^ [34,35,36,37]. Based on the above excellent photo-physical properties, in this work, we design a new cyan fluorescence probe **1**, which was composed of the ICT-based fluorophore, 4′-hydroxybiphenyl-4-carbonitrile and *N, N*-dimethylthiocarbamate group for the selectivity detection of ClO^−^. Probe **1** is nearly non-fluorescent because of the quenching effect of the *N, N*-dimethylthiocarbamate group. However, upon the treatment of ClO^−^, probe **1** can be rapidly converted to dye **2**, which has been verified by high-resolution mass spectrometry (Figure S9). Therefore, the ICT process from the electron-donating group (hydroxyl moiety) to the electron-withdrawing group (cyano moiety) in probe **1** is recovered, which released a pronounced cyan fluorescence signal. As expected, probe **1** displays a satisfactory sensitivity toward ClO^−^ with the low detection limit (72 nM). Attractively, with the aid of probe **1**, we effectively monitor the fluctuation of the ClO^−^ level in living MCF-7 cells and HeLa cells as well as successfully imaged endogenous ClO^−^ in a mouse model of fluoxetine-induced liver injury.

## 2. Results and Discussion

### 2.1. Spectral Properties of Probe ***1*** towards ClO^−^

**Figure 1:**
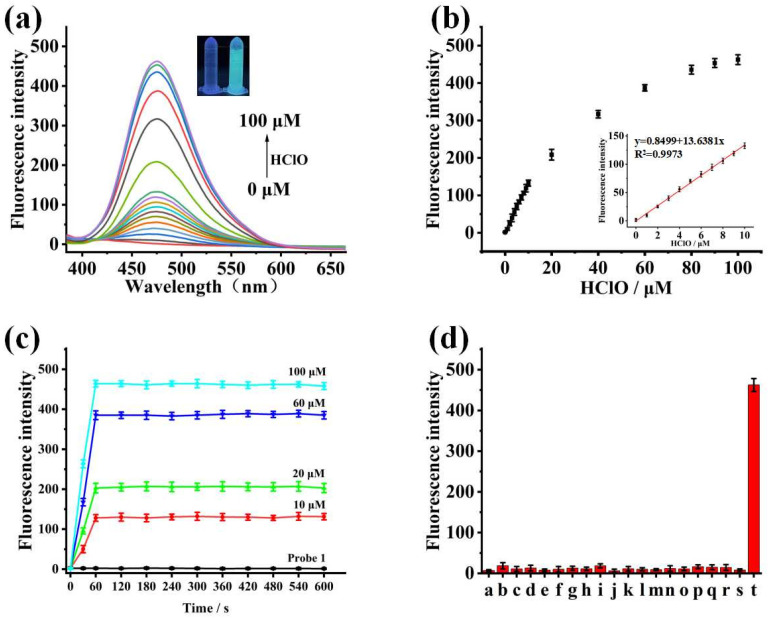
(**a**) Changes in fluorescence spectra of probe **1** (10 μM) in response to different concentrations of ClO^−^ (0–100 μM). (**b**) The linearity between the fluorescence intensity (475 nm) and the concentration of ClO^−^. (**c**) Time-dependence fluorescence responses of 10 μM probe **1** toward various concentrations of ClO^−^ (0, 10, 20, 60, 100 μM) in PBS buffer (pH 7.4, 40% DMSO, *v*/*v*). (**d**) Fluorescence response of probe **1** (10.0 µM) with 100 μM bio-analytes (a–t: (a) probe, (b) ONOO^–^, (c) H_2_O_2_, (d) ·O^t^Bu, (e) TBHP, (f) NO, (g) O_2_^·−^, (h) •OH, (i) Cu^2+^, (j) Na^+^, (k) Mg^2+^, (l) Ca^2+^, (m) HS^−^, (n) HCO_3_^−^, (o) SO_4_^2−^, (p) NO_2_^−^, (q) Hcy, (r) Cys, (s) GSH, (t) ClO^−^).

With probe **1** in hand, we firstly investigated its recognition ability to ClO^−^ by UV–vis spectroscopy. As shown in Figure S1 (Supplementary Materials), free probe **1** showed a maximum absorption peak at 280 nm. After the treatment of ClO^−^, new absorption peaks at 350 nm generated and increased sharply, which corresponded to the absorption characteristics of dye **2**. In the fluorescence spectra, probe **1** exhibited almost non-fluorescent emission at measured wavelengths, while upon the addition of a certain concentration (0–100 μM) of ClO^−^, there was a gradual enhancement of fluorescence intensity at 475 nm under the same excitation and a prominent color change from colorless to cyan observed facilely (Figure 1a). Simultaneously, the fluorescence intensity showed a good linear (R^2^ = 0.9973) response to added ClO^−^, and the LOD (LOD = 3S_0_/K) was calculated to be 72 nM (Figure 1b). The optical inspection manifested probe **1** had good response features for ClO^−^ detection.

### 2.2. Response Time and pH Effects of Probe ***1*** towards ClO^−^

Time-dependence was a significant index for the reaction-based fluorescence probe. It was found that the response speed of the probe **1** was fairly fast to various amounts of ClO^−^ (10, 20, 60.0, 100 μM), which was completed within 60 s (Figure 1c). Moreover, probe **1** itself possessed apparently stable and weak fluorescence at the same reaction time. In view of using in biological vectors, the pH effect on the fluorescence enhancement of probe **1** was crucial to biological applications. Thus, the fluorescence signal of probe **1** toward ClO^−^ with different pH values was also investigated. The data demonstrated that probe **1** could sensibly perceive the presence of ClO^−^ over a broad pH value scale of 6–8 (Figure S2). At the same time, probe **1** can maintain the original state. The test results displayed that probe **1** had the potential to become a valid tool for the detection of ClO^−^ in a microenvironment at a neutral pH value of 7.4.

### 2.3. Selectivity and Competitiveness of Probe ***1*** toward ClO^−^

In light of the complex internal environment of biological systems, it was necessary to study the specificity of the probe toward ClO^−^. Thus, a variety of bio-related species (including (a) blank (b–s), (b) ONOO^–^, (c) H_2_O_2_, (d) ·O^t^Bu, (e) TBHP, (f) NO, (g) O_2_^·−^, (h) •OH, (i) Cu^2+^, (j) Na^+^, (k) Mg^2+^, (l) Ca^2+^, (m) HS^−^, (n) HCO_3_^−^, (o) SO_4_^2−^, (p) NO_2_^−^, (q) Hcy, (r) Cys, (s) GSH, and (t) ClO^−^) were measured. As observed in Figure 1d, only ClO^−^ could give rise to a remarkable increase in the fluorescence intensity of probe **1** at 475 nm, while other tested bio-analytes hardly induced fluorescence intensity changes. To demonstrate the anti-interference feasibility of probe **1** in a complex internal environment, a diverse array of competitive experiments with representative biorelevant species were established (Figure S3). First, probe **1** was treated with ClO^−^ in the presence of various types of interfering substances, such as thiol (Cys, Hcy and GSH), cations (Na^+^, Cu^2+^, Mg^2+^, Ca^2+^), anions (HS^−^, HCO_3_^−^, SO_4_^2−^, NO_2_^−^) and reactive oxygen species (ONOO^−^, H_2_O_2_, ·O^t^Bu, TBHP, NO, O_2_^·−^, •OH). There was no noticeable interference for probe **1** to detect ClO^−^ with the coexistence of other bio-analytes, confirming that probe **1** possessed a latent capacity for monitoring ClO^−^ efficiently and sensitively in vivo (Figure S3).

### 2.4. Response Mechanism Study

**Scheme 1:**
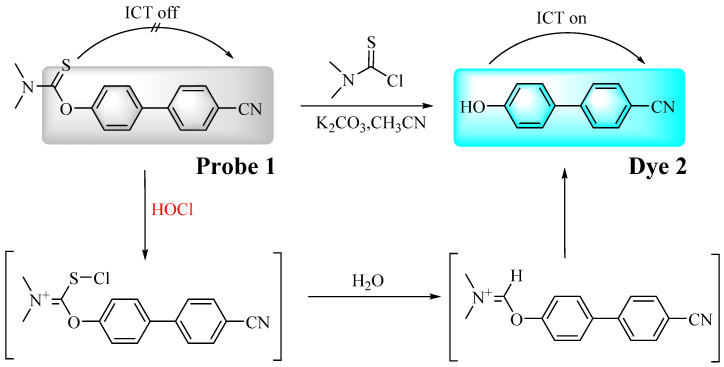
The synthesis route of probe **1** and recognition mechanism of HClO.

The response mechanism study of probe **1** toward ClO^−^ could be reasonably illustrated by Scheme 1. Due to its excellent push–pull electronic system and enormous Stokes shift, as an excellent fluorescent reporter, 4′-hydroxybiphenyl-4-carbonitrile has been reported to show outstanding optical properties in the development of small molecule fluorescent probes [38]. According to the previous research results, we rationally chose the 4′-hydroxybiphenyl-4-carbonitrile (dye **2**) with a large Stokes shift as the luminophore, and we selected the *N, N*-dimethylthiocarbamate group as the response group. Eventually, a ClO^−^ responsive fluorescent probe **1** was obtained, and this new fluorescent probe **1** showed non-fluorescence in the cyan region because of the ICT process inhibition, which was caused by the *N, N*-dimethylthiocarbamate group. Upon reaction with ClO^−^, the *N, N*-dimethylthiocarbamate group in probe **1** was easily attacked by HOCl, and the production of intermediate immonium was quickly induced by Cl^+^ from the decomposition of HOCl, which was followed by the attack of Cl^+^ leading to unstable formate ester after the process of hydrolysis. Finally, dye **2** was released from the sequent process of hydrolysis. Correspondingly, the tested solution obtained a remarkable fluorescent signal in the cyan region because of the recovery of the ICT process from the electron-donating group (hydroxyl moiety) to the electron-withdrawing group (cyano moiety) in probe **1**, and these optical properties allowed probe **1** to be applied for the sensitive monitoring of ClO^−^ with an intense cyan signal. To further confirm this sensing mechanism, we tested the fluorescent product of probe **1** with ClO^−^ by using HRMS and NMR analysis. The fluorescent product of probe **1** with ClO^−^ exhibited a quite similar ^1^H NMR and ^13^C NMR spectrum to that of dye **2** (Figures S10 and S11). Additionally, as indicated in Figure S8, the mass peak of probe **1** was founded at *m*/*z* = 283.0888 (calcd. for [M + H]^+^: 283.0905). In contrast, when the mixture of probe **1** and ClO^−^ was recorded by a high-resolution mass spectra (HRMS), a novel peak at *m*/*z* =194.0611 was collected (Figure S9), corresponding to dye **2** (calcd. for [M − H]^+^: 194.0611). According to the above outcomes, the hypothetical reaction mechanism of free probe **1** for detecting ClO^−^ obtained a more reasonable explanation.

### 2.5. Cellular Imaging Experiment

**Figure 2:**
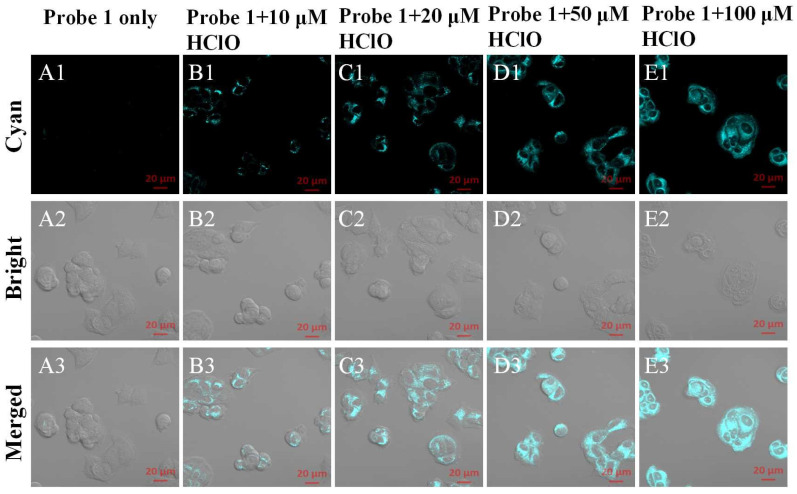
Bio-imaging of exogenous ClO^−^ in MCF-7 cells. (**A1**–**A3**) Only treated with probe **1** (10 μM) for 30 min; (**B1**–**B3**,**C1**–**C3**,**D1**–**D3**,**E1**–**E3**) incubated with probe **1** (10 μM) for 30 min and then treated with different concentrations of ClO^−^ (10, 20, 50, 100 μM) for another 30 min. (λ_ex_ = 405 nm, λ_em_ = 460–510 nm). Scale bars = 20 μm.

The feasibility of probe **1** for sensing the exogenous ClO^−^ level in MCF-7 cells was assessed. Firstly, the study on the cytotoxicity of probe **1** was conducted by the traditional MTT method. It was worth noting that probe **1** held low toxicity to the living cells; even though the concentration reaches as high as 50 μM, the cell viability was more than 90% (Figures S4 and S5). Then, the exogenous ClO^−^ was imaged in MCF-7 cells with laser scanning confocal microscopy. When MCF-7 cells were loaded in probe **1**, negligible fluorescence enhancement was captured at the cyan channel (Figure 2A). In contrast, obvious fluorescence was acquired by being incubated with ClO^−^ and probe **1**. What is more, the fluorescence intensity in the cyan channel was directly proportional to the addition of ClO^−^ (Figure 2B–E). The above outcomes demonstrated that probe **1** was able to map the ClO^−^ level in MCF-7 cells.

**Figure 3:**
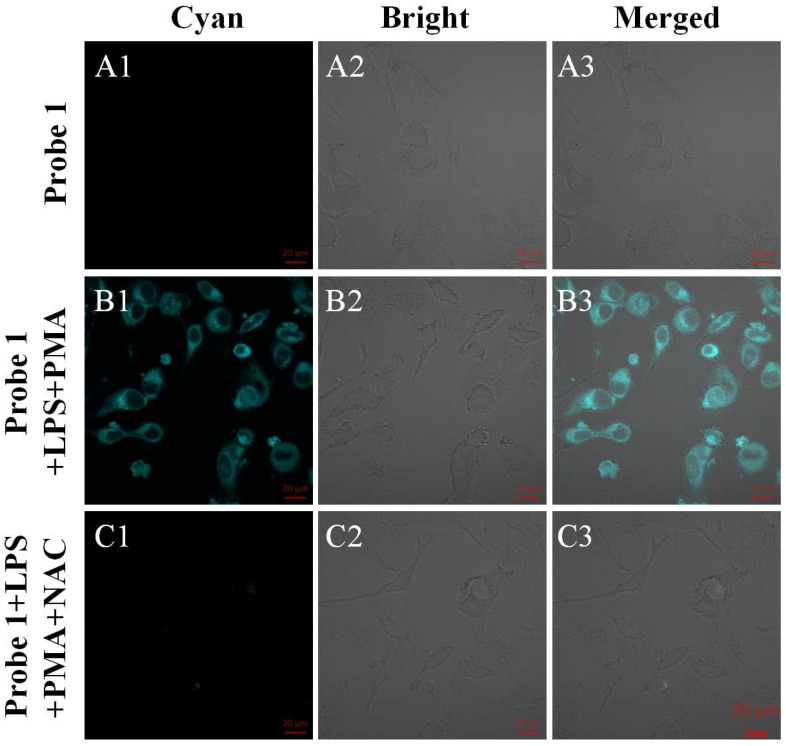
Fluorescence images of ClO^−^ in HeLa cells. (**A1**–**A3**) only probe **1**-loaded; (**B1**–**B3**) pre-stimulated with LPS (0.5 μg/mL) and PMA (1 μg/mL), followed by probe **1** staining; (**C1**–**C3**) pre-treated with NAC (200 μM) before stimulation with LPS and PMA, then incubated with probe **1**. The cyan channel (460–510 nm) was excited at 405 nm. Scale bar: 20 μm.

Considering the response performance of probe **1** in aqueous solution, we further explored the capability of probe **1** for intracellular ClO^−^ in HeLa cells. As depicted in Figure 3, compared with probe **1** alone (Figure 3A), the treatment of LPS (lipopolysaccharide, a common endotoxin) and PMA (phorbol 12-myristate 13-acetate, commonly used to upregulate endogenous ClO^−^ levels) released a significantly cyan fluorescence signal, revealing that a certain concentration of ClO^−^ was present in HeLa cells. Meanwhile, when HeLa cells were pre-treated with NAC (N-acetylcysteine, a HClO scavenger) (Figure 3C), the cyan channel displayed a weak fluorescence signal, since the upregulated endogenous ClO^−^ levels could be competently depressed by NAC. These conclusions suggested that probe **1** could hold great promise for visualizing ClO^−^ in living cells. In the meantime, these gratifying experimental results also laid the foundation for imaging endogenous ClO^−^ in organisms.

### 2.6. Confocal Fluorescence Imaging in Mice Liver Tissue

**Figure 4:**
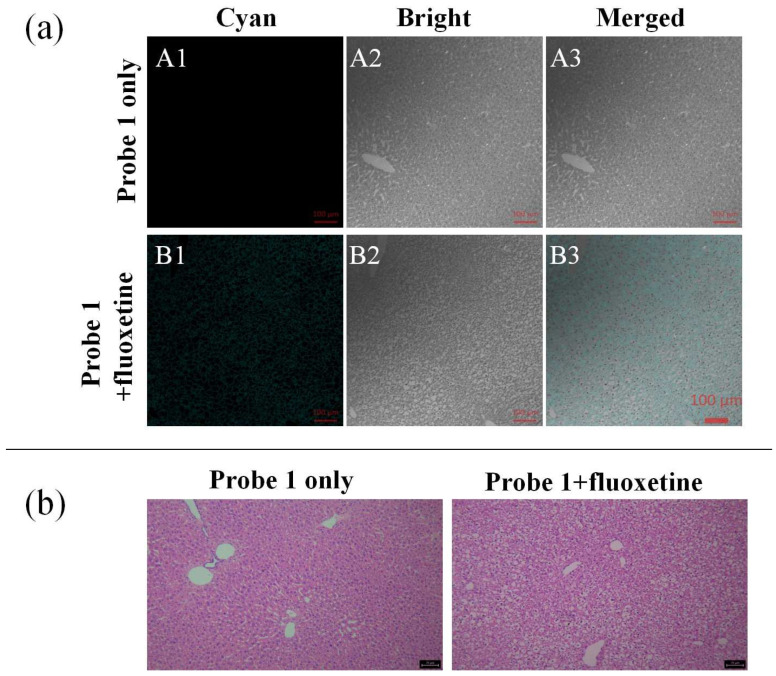
(**a**) Confocal fluorescence imaging of fluoxetine-induced ClO^−^ in mice liver tissue. (**A1**–**A3**) incubation of probe **1** (100 µM); (**B1**–**B3**) pre-treatment of fluoxetine (100 mg/kg) and upon addition of probe **1** (100 µM). (**b**) Microscopy images of hematein and eosin dyed liver sections of the mice with different treatments (probe **1** alone, probe **1** + fluoxetine). Emissions were collected at 460–510 nm for the cyan channel (excitation wavelength: 405 nm). Scale bar: 100 µm.

As known to all, depression was one of the most common mental illnesses, which led to an increased risk of suicide, resulting in serious socioeconomic consequences. In the course of clinical diagnosis and treatment, a variety of antidepressant drugs had been developed and widely used. Nevertheless, the improper intake of antidepressant drugs could also inevitably cause drug induced liver injury (DILI). Evidence was mounting that ClO^−^ had been verified as a potential biomarker in DILI. Inspired by the above performance, we applied probe **1** to monitor the upregulation of ClO^−^ level in the DILI model. Fluoxetine was a common antidepressant drug and usually was unfortunately taken for a long time, and the mechanism of hepatotoxicity caused by fluoxetine was not well understood. Accordingly, it was necessary to study the hepatotoxicity caused by fluoxetine at the tissue level. As illustrated in Figure 4a, there was no cyan fluorescence signal detected in the liver tissue of the control mice, indicating a low concentration of ClO^−^ in normal liver tissue. Consistent with the outcomes in HeLa cells and zebrafish, when mice were treated with fluoxetine (100 mg/kg), a bright fluorescence in the cyan channel was acquired, meaning upregulated ClO^−^ appears in the damaged liver after the stimulation of fluoxetine. Finally, the histological analysis on liver tissues was carried out to further clarify liver injury during fluoxetine administration. In a short, mice liver slices were prepared from two groups processed with different conditions, containing probe **1** and fluoxetine administration. As shown in Figure 4(A1), the hepatocytes of the control group (probe **1** only) were densely arranged around the central vein, and the nucleus of the hepatocytes were structurally normal. Compared with probe **1**-stained alone (Figure 4b), the apparent histological changes (including the ballooning degeneration of liver cells and disorder of liver plates) were observed by fluoxetine administration, implying the serious liver injury of mice. The data of confocal fluorescence imaging and histological analysis on liver tissues indicated that probe **1** could availably detect fluoxetine-induced ClO^−^ in liver injury and be used as a reliable sensor for the early warning of DILI.

## 3. Experimental

### 3.1. Instruments and Reagents

Unless otherwise noted, all the reagents were purchased from commercial suppliers and were used without additional purification ^1^H NMR and ^13^C NMR spectra were recorded on a Bruker Avance 600 MHz spectrometer. Imaging of cells and mice liver tissues was performed with a Zeiss LSM710 Wetzlar (Oberkochen, Germany) laser scanning confocal microscope. High-resolution mass spectra data were obtained by AB Sciex TripleTOF 4600. UV-vis absorption spectra were collected through a U-3900 UV-vis spectrometer (Hitachi, Tokyo, Japan). Fluorescence spectra were recorded with a HITACHI F-4600 fluorescence spectrophotometer.

### 3.2. Preparation of Various ROS and RNS Solutions

Hypochlorous acid (HClO) solution was achieved by diluting commercial sodium hypochlorite solution. Peroxynitrite (ONOO^–^), •OH and TBHP were prepared according to the literature method [39]. ·O^t^Bu, Superoxide anion (O_2_^•–^), and nitric oxide (NO) were acquired following the previous reporting method [40].

### 3.3. Spectrophotometric Measurements

Fluorescence spectra were measured in a 1.0 cm quartz sampling cell. The stock solution of 0.3 mM was prepared by dissolving probe **1** in DMSO. A concentration of 10.0 mM of various analytes (ONOO^–^, H_2_O_2_, ·O^t^Bu, TBHP, NO, O_2_^·−^, •OH, Cu^2+^, Na^2+^, Mg^2+^, Ca^2+^, HS^−^, HCO_3_^−^, SO_4_^2−^, NO_2_^−^, Hcy, Cys, GSH, HClO) was used for the selectivity analysis. All the test solution was placed in 3.0 mL of DMSO-PBS solution (4:6, *v*/*v*, pH = 7.4) including the diluted probe **1** stock solution (10 μM) and an appropriate concentration of different analytes. For the monitoring of ClO^−^ and other test substances, the solution was mixed at 25 °C for 30 s, and then, the fluorescence spectra were collected at λ_ex/em_ = 340/475 nm. Slit width: 5/5 nm.

### 3.4. Cell Culture and MTT Viability Assays

HeLa cells and MCF-7 cells were inoculated in Dulbecco’s modified Eagle’s medium (DMEM) supplemented with 10% fetal bovine serum, 1% penicillin, and 1% streptomycin, and grown at 37 °C in the 5% CO_2_ environment. Before imaging experiment, cells were separately inoculated and plated on laser confocal culture dishes for 12 h at 37 °C in the 5% CO_2_ environment. The cytotoxicity of probe **1** was investigated by the classical MTT method [41]. HeLa cells and MCF-7 cells were performed in a 96-well plate and further incubated with different concentrations of probe **1** (0, 10, 20, 30, 40, 50 μM) for 24 h at 37 °C.

### 3.5. Synthesis of Probe ***1***

Dye **2** (117.2 mg, 0.6 mmol) and dimethylthiocarbamoyl chloride (73.79 mg, 0.6 mmol) were dissolved in dichloromethane (10.0 mL). Anhydrous potassium carbonate (82.92 mg, 0.6 mmol) was added to the solution, and the mixture was refluxed for 5 h at 83 °C. After the reaction was completed, the reaction solution was extracted with dichloromethane (3 × 10 mL), and the organic layer was collected and dried over anhydrous Na_2_SO_4_. The solvent was removed under reduced pressure, and the crude product was further purified by chromatography on silica gel using petroleum ether/ethyl acetate, *v*/*v* = 2:1, as an eluent to obtain a white solid product (yield, 74%). ^1^H NMR (600 MHz, DMSO) δ 7.92 (dd, J = 19.0, 8.4 Hz, 4H), 7.79 (d, J = 8.5 Hz, 2H), 7.21 (d, J = 8.5 Hz, 2H), 3.38 (s, 3H), 3.34 (s, 3H).^13^C NMR (151 MHz, DMSO) δ 185.95, 154.11, 143.66, 135.40, 132.73, 127.35, 123.41, 118.70, 109.85, 42.69, 38.41.HRMS (EI) *m*/*z* calcd for [C_16_H_14_N_2_OS + H]^+^: 283.0905, Found: 283.0888.

### 3.6. Exogenous and Endogenous ClO^−^ Imaging

For exogenous ClO^−^ imaging, MCF-7 cells were treated with probe **1** (10 μM) for 30 min as a control group. The other MCF-7 cells groups were cultured in advance with different concentrations of ClO^−^ (10, 20, 50, 100 µM) for 30 min, and then, they were treated with probe **1** (10 µM) for another 30 min. After washing with PBS, the images were collected using confocal microscopy. In order to obtain endogenous ClO^−^, HeLa cells were selected and stimulated with lipopolysaccharide (LPS) (1 μg/mL) for 12 h and Phorbol-12-Myristate-13-Acetate (PMA) (1 μg/mL) for 1 h; then, they were loaded with probe **1** (10 μM) for 30 min. Other HeLa cells were pre-loaded with N-acetylcysteine (NAC) before the stimulation of LPS and PMA, which was followed by staining with probe **1**. The control group was only incubated with probe **1**. The treated cells were imaged with 405 nm excitation and 460–510 nm collection.

### 3.7. Confocal Imaging in Mice Liver Tissues

An animal model of the liver injury was built through the intraabdominal injection of fluoxetine in Kunming mice (~30 g) according to the reported studies [42]. All the studies were performed according to the guidelines by the Animal Experimentation Ethics Care Committee of Harbin University of Commerce (HSDYXY-20200307). Mice injected with fluoxetine (100 mg/kg) were cultivated overnight and subsequently stained with probe **1** (100 µM, 100 µL) via the tail vein for 30 min. The established-model mice were dissected to collect the livers, which were cut into slices for confocal imaging. The mice in a control group underwent probe **1** (100 µM, 100 µL) alone-injected via the caudal vein. The image data were recorded at the cyan channel with 405 nm excitation.

## 4. Conclusions

In summary, we have successfully developed a cyan emission probe **1** for sensitively detecting ClO^−^, which exhibited a low detection limit (72 nM), high selectivity, wide pH operating range, great tissue penetration and low auto-fluorescence. In the opinion of the merits, probe **1** can be potentially applied for the monitoring of exogenous ClO^−^ in living MCF-7 cells. In addition, probe **1** has been employed to image and detect endogenous ClO^−^ levels in living HeLa cells and demonstrated its powerful imaging capabilities and high degree of specificity in the complex internal environment of living cells. More importantly, probe **1** has been successfully applied to the established fluoxetine-induced liver injury model, and it also confirmed that ClO^−^ could be over-expressed in the process of drug-induced liver injury. The above results displayed that the probe **1** would be a useful tool for the development of liver protection drugs and the investigation of pathological roles of ClO^−^ in disease and the early diagnosis of DILI.

## Data Availability

All data generated or analyzed during this study are included in this published article (and its Supplementary Information Files) or are available from the corresponding author on reasonable request.

## Supplementary Materials

The following supporting information can be downloaded at: https://www.mdpi.com/article/10.3390/bios13070744/s1, Table S1: Comparison of fluorescent probes for ClO^−^; Figure S1: The absorption spectra of 10 μM probe **1** (red), dye **2** (black) and probe **1** reacted with 100 μM ClO^−^ (blue); Figure S2: The fluorescence intensity of probe **1** (10.0 μM) before (■) and after (●) incubating with ClO^−^(100.0 μM) at different pH value (2.0–11.0); Figure S3: Fluorescence intensity (475 nm) of probe **1** (10.0 μM) after incubating with HClO (100.0 μM) in the presence of different competition species. (a) ONOO^–^, (b) H_2_O_2_, (c) ·O^t^Bu, (d) TBHP, (e) NO, (f) O_2_^·−^, (g) •OH, (h) Cu^2+^, (i) Na^+^, (j) Mg^2+^, (k) Ca^2+^, (l) HS^-^, (m) HCO_3_^−^, (n) SO_4_^2−^ (o)NO_2_^−^, (p) Hcy, (q) Cys (r) GSH; Figure S4: Cytotoxicity assays of probe **1** at different concentrations (0.0, 10.0, 20.0, 30.0, 40.0, 50.0 μΜ) for HeLa cells; Figure S5: Cytotoxicity assays of probe **1** at different concentrations (0.0, 10.0, 20.0, 30.0, 40.0, 50.0 μΜ) for MCF-7 cells; Figure S6: ^1^H NMR spectrum of probe **1** in DMSO-*d_6_*; Figure S7: ^13^C NMR spectrum of probe **1** in DMSO-*d_6_*; Figure S8: HRMS spectrum of probe **1**; Figure S9: HRMS spectrum of probe **1** + ClO^−^; Figure S10: ^1^H NMR spectrum of the fluorescent product of probe **1** with ClO^−^ in DMSO-*d_6_*; Figure S11: ^13^CNMR spectrum of the fluorescent product of probe **1** with ClO^−^ in DMSO-*d_6_*.
