# Supplementary material for: A Simple ICT-Based Fluorescent Probe for HOCl and Bioimaging Applications
Source: Biosensors (Basel). 2023 Jul 18;13(7):744. doi: 10.3390/bios13070744 (PMC10377358; doi:10.3390/bios13070744)
Supplement: Supplementary file 1 [file biosensors-13-00744-s001.zip › biosensors-2469518-supplementary.pdf]

Supporting information for

A simple ICT-based fluorescent probe for HOCl and bioimaging applications

Yan Zheng [a], Shuang Wu[a], Yifan Bing[a], Huimin Li[a], Xueqin Liu[a], Wenlan Li[a,b], Xiang Zou[b*], Zhongyuan Qu[a*]

[a]School of Pharmacy, Harbin University of Commerce, Harbin 150076, China.

[b]Engineering Research Center on Natural Antineoplastic Drugs, Ministry of Education,

Harbin University of Commerce 150076, China.

* *Corresponding author.*
E-mail address: zouxiang@hrbcu.edu.cn; qiuqiuqu@163.com

Table of contents

Page

Table S1..S1

Figures S1-S2..S3

Figures S3-S4..S4

Figures S5-S6..S5

Figures S7-S8..S6

Figures S9-S10..S7

Figure S11...S8

Table S1. Comparison of fluorescent probes for ClO^-

Probe	Stokes shift	Test system	Detection limit	Response time	Application	Literature
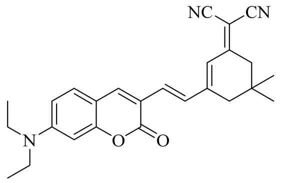	155 nm	PBS buffer (pH7.4, containing 50% DMSO）	0.17 μM	100 s	HepG2 cells Mice	Sensors & Actuators: B. Chemical, 2019, 287, 453-458
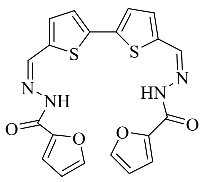	105 nm	Bis-tris buffer (10 mM, pH = 7.4)	4.2 μM	--	Zebrafish	Spectrochim. Acta A Mol. Biomol. Spectrosc, 2021, 261, 120059
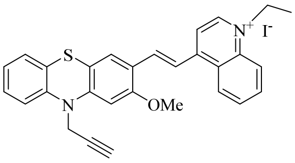	65 nm	PBS buffer (pH 7.4, 10 mM, 20% DMSO)	58 nM	12 s	HeLa cells	Spectrochim. Acta A Mol. Biomol. Spectrosc, 2021, 246, 118953
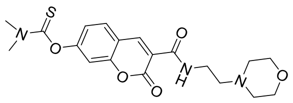	106 nm	PBS buffer (10 mM, pH 7.4, 10% DMF)	24.3 nM	25 s	HeLa cells	Anal. Chim. Acta, 2020, 1094, 122-129
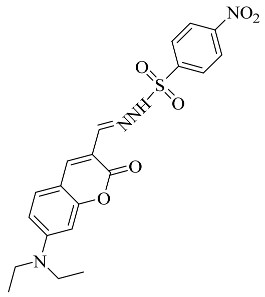	55 nm	PBS (pH = 7.4, containing 5 % MeOH)	0.355 μM	30 s	RAW 264.7 Cell, Zebrafish mouse liver slice	Journal of Photochemistry & Photo-biology, A:Chemistry, 2023,434, 114204

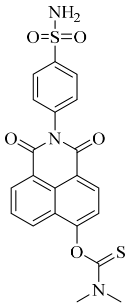	100 nm	PBS buffer (10 mM, pH 7.4, 5% CH_3CN)	57 nM	3 s	HeLa cells	Spectrochimica Acta Part A: Molecular and Biomoleculr Spectroscopy 2023,286, 121986
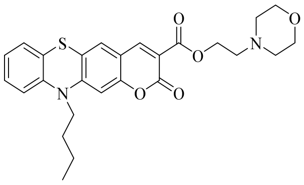	75 nm	PBS (10.0 mM, pH 7.4, containing 1% CTAB)	0.58 μM	60 s	RAW 264.7 cell Zebrafish	Spectrochimica Acta Part A: Molecular and Biomoleculr Spectroscopy 2021, 261, 120024
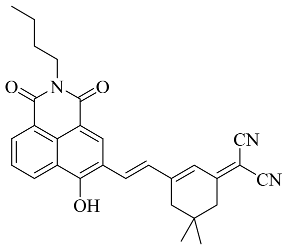	205 nm	PBS buffer (10.0 mM, pH7.4, containing 5% CH_3CN)	0.738 μM	3 s	HeLa cells,	Anal. Methods, 2021, 246, 118960
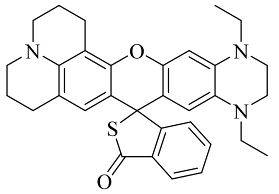	84 nm	PBS buffer (20 mM, pH7.4, containing 50% acetonitrile)	74 nM	100 s	HeLa cells, Zebrafish	Tetrahedron, 2022, 127, 133020
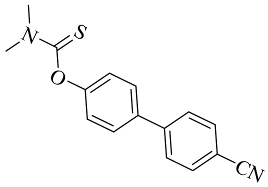	**125 nm**	**PBS buffer (pH7.4, 20 mM, containing 40% DMSO）**	**72 nM**	**30 s**	**HeLa cells MCF-7 cells Mice liver tissue**	**This work**

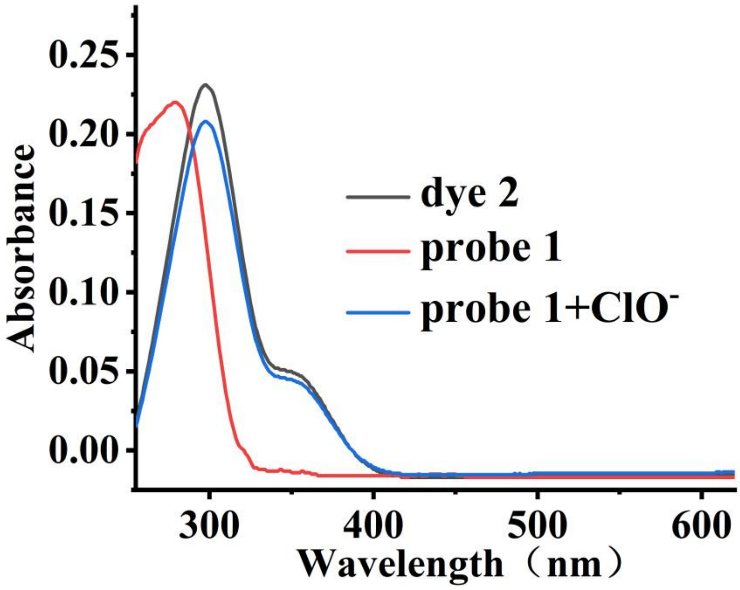

Figure S1. The absorption spectra of 10 μM probe **1** (red), dye **2** (black) and probe **1** reacted with 100 μM ClO^- (blue).

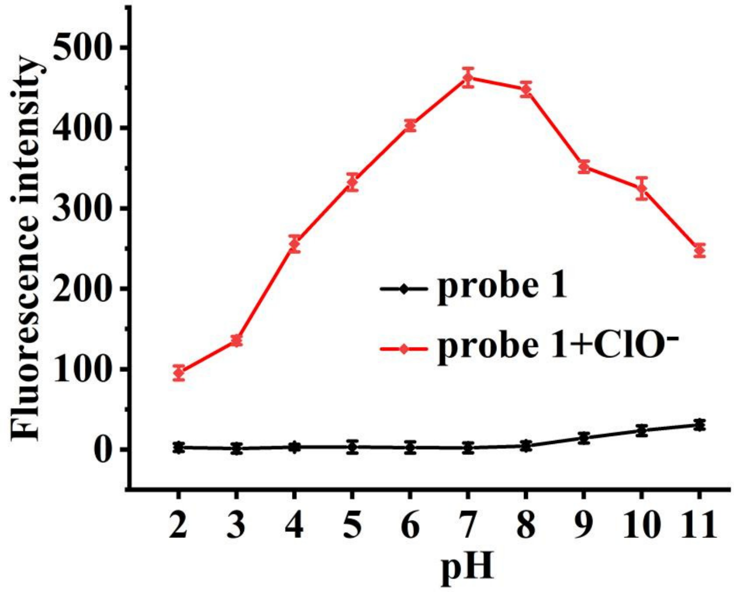

Figure S2. The fluorescence intensity of probe **1** (10.0 μM) before (■) and after (●) incubating with ClO^-(100.0 μM) at different pH value (2.0-11.0).

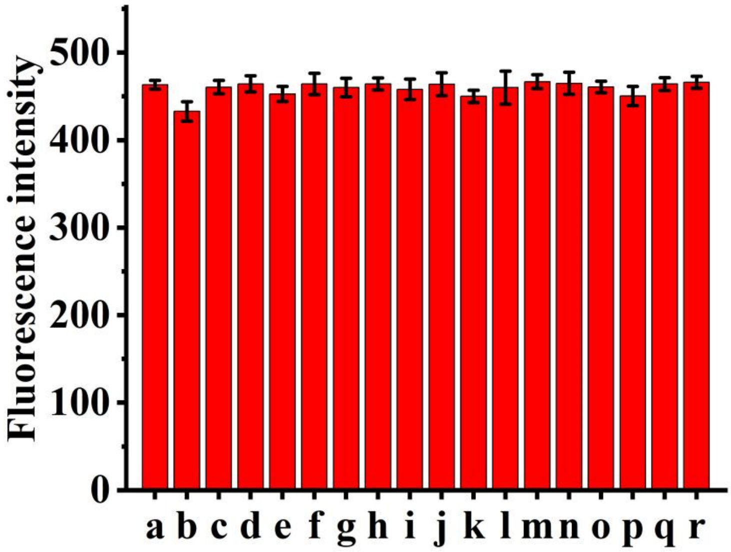

Figure S3. Fluorescence intensity (475 nm) of probe **1** (10.0 μM) after incubating with HClO (100.0 μM) in the presence of different competition species. (a) $ONOO^{-}$, (b) H_2O_2, (c) $\cdot O^tBu$, (d) TBHP, (e) NO, (f) $O_2^{\cdot -}$, (g) •OH, (h) Cu^{2+}, (i) Na^{+}, (j) Mg^{2+}, (k) Ca^{2+}, (l) HS^{-}, (m) HCO_3^{-}, (n) SO_4^{2-} (o)NO_2^{-}, (p) Hcy, (q) Cys (r) GSH.

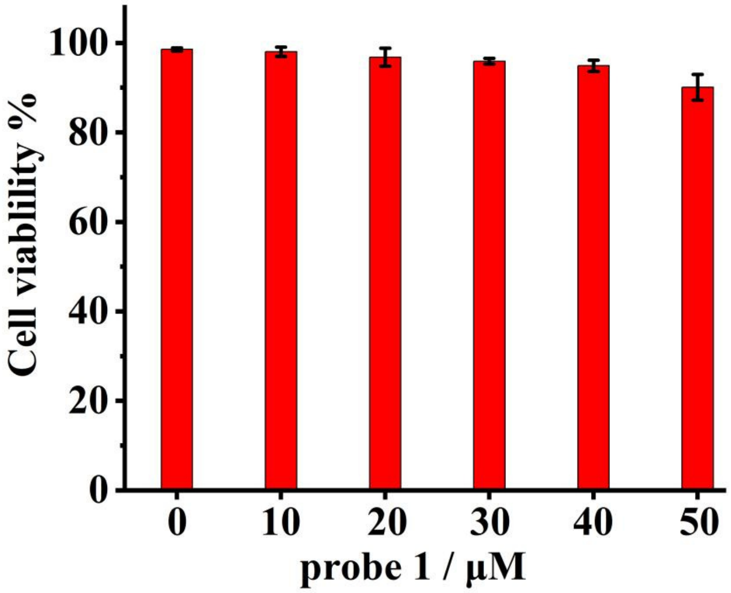

Figure S4. Cytotoxicity assays of probe **1** at different concentrations (0.0, 10.0, 20.0, 30.0, 40.0, 50.0 μM) for HeLa cells.

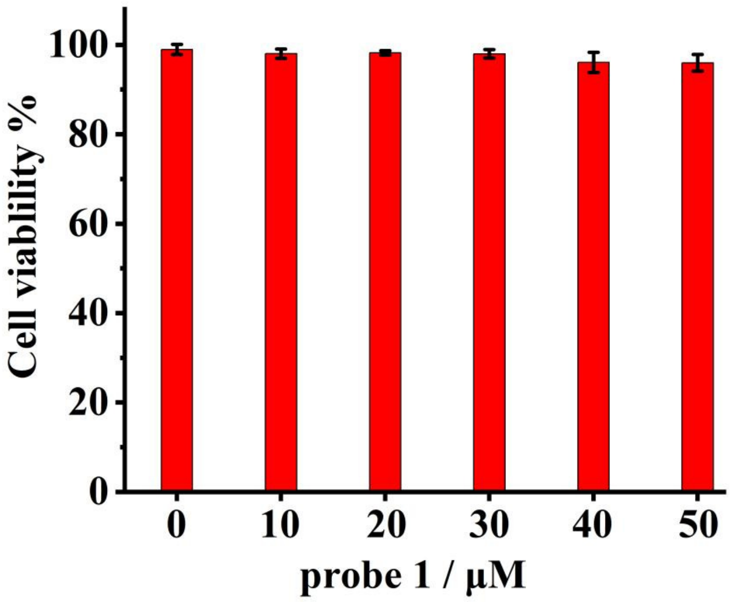

Figure S5. Cytotoxicity assays of probe **1** at different concentrations (0.0, 10.0, 20.0, 30.0, 40.0, 50.0 μM) for MCF-7 cells.

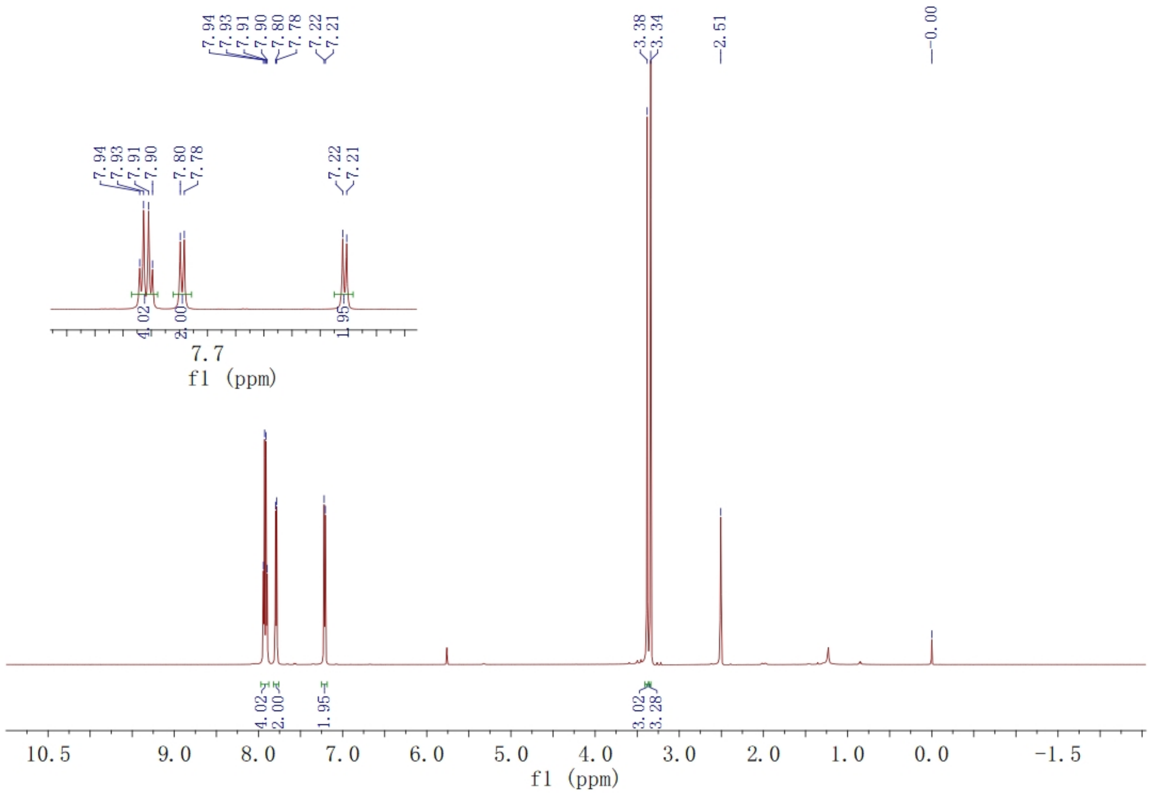

Figure S6. ^{1}H NMR spectrum of probe **1** in DMSO-*d₆*.

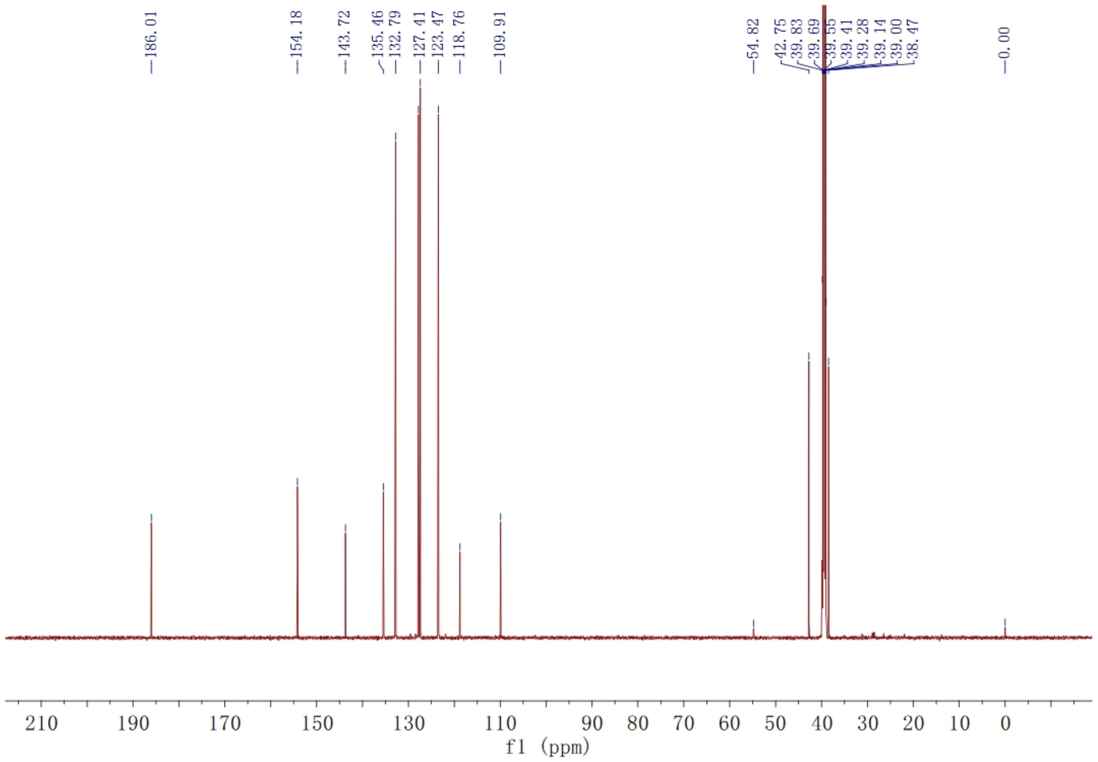

Figure S7. ^{13}C NMR spectrum of probe 1 in DMSO-d_6.

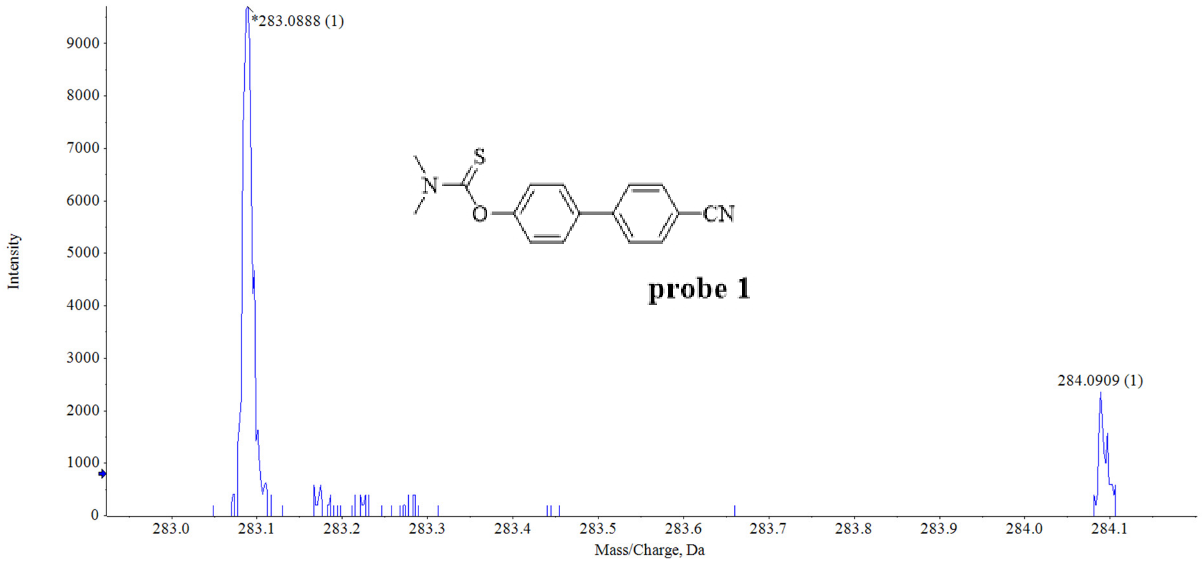

Figure S8. HRMS spectrum of probe **1**.

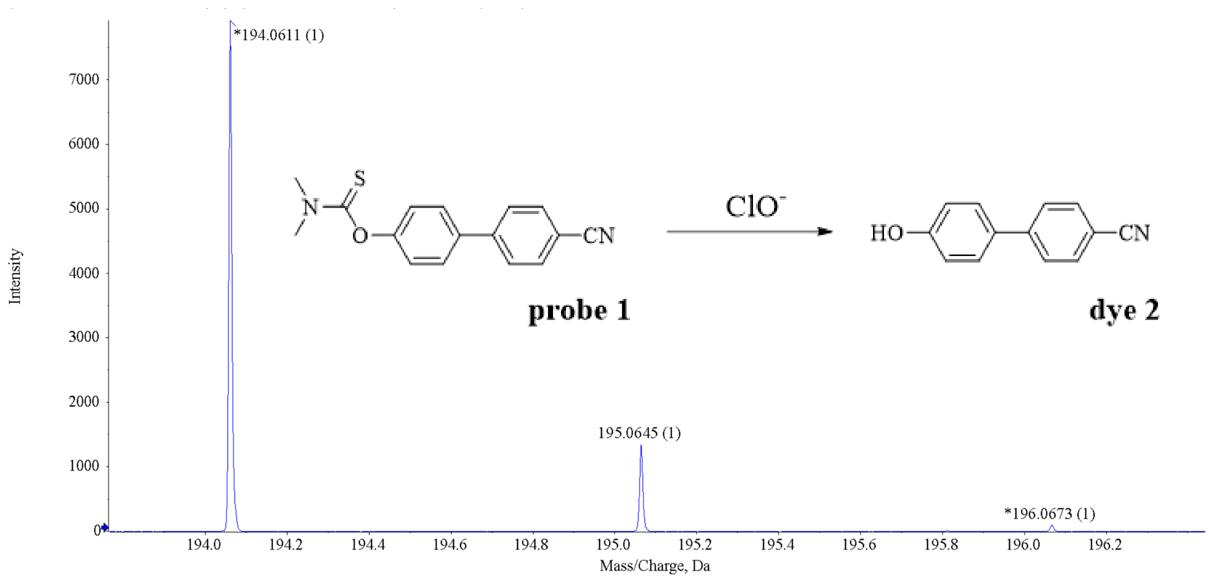

Figure S9. HRMS spectrum of probe **1** + ClO^-.

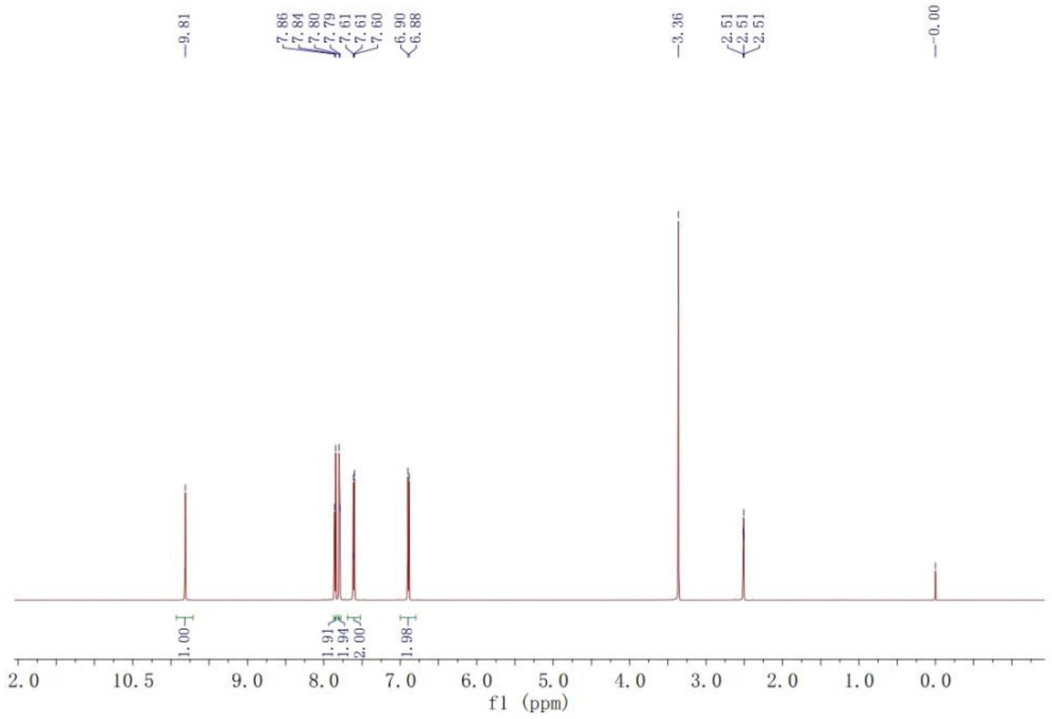

Figure S10. ^1H NMR spectrum of **t**he fluorescent product of probe 1 with ClO^- in DMSO-d_6.

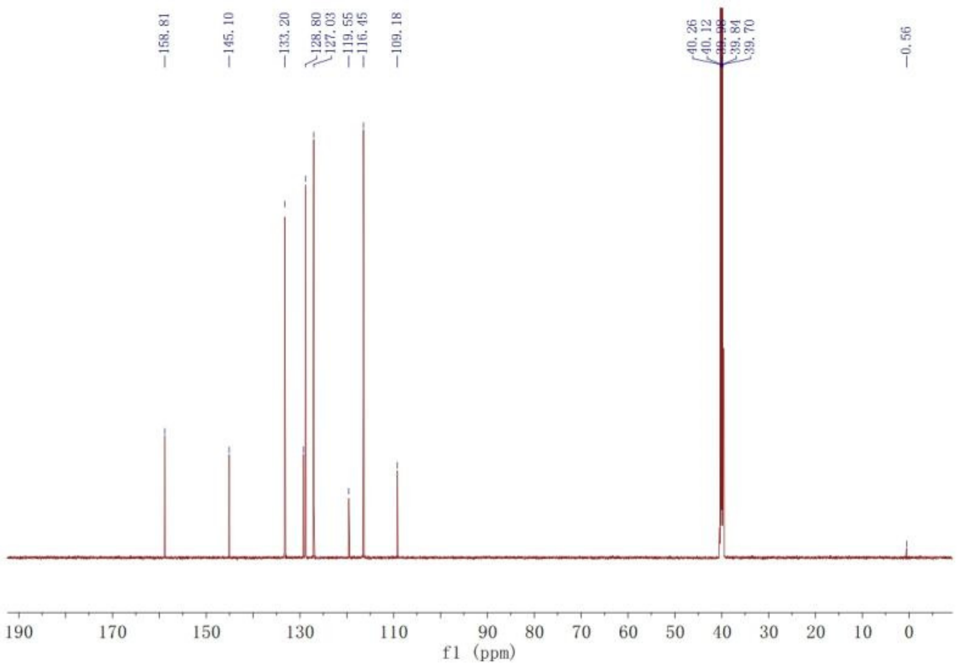

Figure S11. ^{13}CNMR spectrum of **t**he fluorescent product of probe 1 with ClO^- in DMSO-d_6.
